# Arteriovenous Malformations (AVMs): Molecular Pathogenesis, Clinical Features, and Emerging Therapeutic Strategies

**DOI:** 10.3390/biom15121661

**Published:** 2025-11-27

**Authors:** Nga Le, Yan Li, Gianni Walker, Bao-Ngoc Nguyen, Arash Bornak, Sapna K. Deo, Omaida C. Velazquez, Zhao-Jun Liu

**Affiliations:** 1 DeWitt Daughtry Family Department of Surgery, University of Miami Miller School of Medicine, Miami, FL 33136, USA; 2 Department of Biochemistry & Molecular Biology, University of Miami Miller School of Medicine, Miami, FL 33136, USA; 3 Department of Surgery, The George Washington University School of Medicine, Washington, DC 20037, USA; 4 Department of Radiology, University of Miami Miller School of Medicine, Miami, FL 33136, USA

**Keywords:** arteriovenous malformation (AVM), molecular signaling, hereditary hemorrhagic telangiectasia (HHT), embolization, stereotactic radiosurgery, vascular diseases

## Abstract

Arteriovenous malformations (AVMs) are fast-flow vascular malformations formed by direct artery-to-vein shunts without an intervening capillary bed, which increases the risk of hemorrhage and organ-specific damage. A synthesis of recent advances shows that AVMs arise from interplay between germline susceptibility (*ENG*, *ACVRL1*, *SMAD4*, *RASA1*, *EPHB4*), somatic mosaicism (*KRAS*, *MAP2K1*, *PIK3CA*), perturbed signaling (TGF-β/BMP, Notch, VEGF, PI3K/AKT, RAS/MAPK), hemodynamic stress, and inflammation. Multimodal imaging—digital subtraction angiography (DSA), MRI/MRA with perfusion and susceptibility sequences, CTA, Doppler ultrasound, and 3D rotational angiography—underpins diagnosis and risk stratification, while arterial spin labeling and 4D flow techniques refine hemodynamic assessment. Management is individualized and multidisciplinary, combining endovascular embolization, microsurgical resection, and stereotactic radiosurgery (SRS); a non-surgical approach and monitoring remain reasonable for some asymptomatic AVMs. Device and technique innovations (detachable-tip microcatheters, pressure-cooker approaches, and newer liquid embolics such as PHIL and Squid) have broadened candidacy, and precision-medicine strategies, including pathway-targeted pharmacotherapy, are emerging for syndromic and somatic-mutation–driven AVMs. Animal models and computational/radiomics tools increasingly guide hypothesis generation and treatment selection. We outline practical updates and future priorities: integrated genomic-imaging risk scores, genotype-informed medical therapy, rational hybrid sequencing, and long-term outcome standards focused on hemorrhage prevention and quality of life.

## 1. Introduction

**Figure 1:**
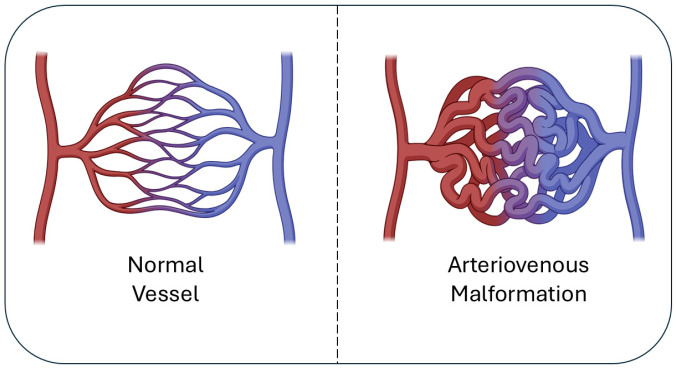
Normal vs. AVM vasculature. Normal vessels (**left**) show orderly artery-capillary-vein connections, while AVMs (**right**) form a tangled nidus with direct artery-vein shunts, leading to high-flow, fragile vessels.

Arteriovenous malformation (AVM) is one form of vascular malformation, which includes arterial, venous, arteriovenous, lymphatic, and combined malformations [1,2,3] (Figure 1). These lesions were first described in the brain by Hubert von Luschka and Rudolf Virchow in the 1850s, laying the foundation for our understanding of cerebrovascular pathology [3,4,5,6]. AVMs are currently identified through Hamburg classification, named and established by a 1988 workshop in Hamburg, Germany, as a clinical guide based on morphological and embryological characteristics [7,8,9]. AVMs are most often congenital in origin, reflecting aberrant vascular development during embryogenesis, although accumulating evidence suggests they may also be dynamic lesions capable of remodeling and progression throughout life under the influence of genetic, inflammatory, and hemodynamic factors [3,4,5]. The clinical consequences of AVMs are determined by size, location, vascular architecture, and flow dynamics. One of the most serious risks is life-threatening hemorrhage, which remains the leading cause of morbidity and mortality in cerebral AVMs (cAVMs) [10]. Hemorrhage may be intraparenchymal, intraventricular, or subarachnoid and often results in long-term neurological deficits. Beyond cAVMs, extracranial AVMs can occur in the gastrointestinal tract, pulmonary vasculature, liver, and musculoskeletal system, where they present with organ-specific dysfunction such as bleeding, high-output heart failure, chronic pain, or soft tissue overgrowth [10,11,12,13]. These diverse manifestations illustrate that AVMs represent a multisystem disease spectrum rather than a purely neurological condition.

The clinical manifestations of AVMs are highly variable. In the brain, they may remain asymptomatic and be discovered incidentally, or they can present with seizures, progressive neurological decline, or acute hemorrhage [14]. Following an initial rupture, the risk of subsequent hemorrhage is considerably higher, underscoring the importance of individualized risk assessment [10,15,16]. Cerebral AVMs are frequently diagnosed in young adults, although pediatric cases often present earlier and may demonstrate more aggressive behavior, particularly when associated with genetic syndromes such as hereditary hemorrhagic telangiectasia (HHT) [4,12,14,17,18,19,20]. In these settings, multiple AVMs may occur across different organ systems, necessitating long-term surveillance and multidisciplinary care [21,22,23].

Recent research has highlighted the molecular underpinnings of AVMs, including germline mutations in *ENG* (endoglin), *ACVRL1* (activin receptor-like kinase 1), and *SMAD4* (Mothers against decapentaplegic homolog 4) in HHT, as well as somatic mutations in *KRAS* (Kirsten rat sarcoma virus), *MAP2K1* (Mitogen-activated protein kinase kinase 1), and *PIK3CA* (phosphatidylinositol-4,5-bisphosphate 3-kinase catalytic subunit alpha) in sporadic lesions [4,18,19,24,25,26,27,28,29,30]. These discoveries have shifted the paradigm from viewing AVMs as static congenital anomalies to considering them as dynamic, genetically influenced vascular malformations amenable to targeted therapies [31,32,33,34]. At the same time, translational advances in imaging, including high-resolution digital subtraction angiography, susceptibility-weighted MRI, arterial spin labeling perfusion imaging, and 4D flow MRI, have greatly enhanced the ability to characterize AVM hemodynamics, identify rupture-prone lesions, and plan interventions [1,2,20,26,35,36,37,38,39,40].

Given their clinical heterogeneity and potential severity, AVMs demand a coordinated, multidisciplinary approach involving neurologists, interventionalists, neurosurgeons, and geneticists [13,21,36]. Parallel progress in preclinical research has provided invaluable insights: animal models in mice and zebrafish have elucidated the contributions of VEGF (vascular endothelial growth factors), Notch, and TGF-β (transforming growth factor–β) signaling, as well as endothelial-to-mesenchymal transition, to AVM initiation and progression [24,41,42]. These models continue to inform therapeutic discovery, particularly for targeted pharmacologic approaches aimed at normalizing vascular structure and function.

Thus, AVMs represent not only a clinical challenge but also a unique biological model at the intersection of vascular biology, genetics, and hemodynamics [43,44]. Their rarity, affecting roughly 1 in 1000 individuals, belies their significant clinical impact, particularly in young patients, and underscores the need for improved strategies in diagnosis and long-term management [11,45]. As research advances, the integration of laboratory discoveries, imaging innovations, and clinical expertise holds promise for more precise and effective care, paving the way toward personalized medicine for AVM patients [3,12,21,31,46].

## 2. Epidemiology and Clinical Presentation

AVMs are relatively uncommon vascular anomalies, with cAVMs being the most extensively studied subtype because of their potential for severe neurological morbidity [29]. There is no definitive sex predominance, though a slight male bias has been observed in some regional studies [47,48]. In pediatric patients, earlier onset and more aggressive behavior are not uncommon, particularly in association with genetic syndromes [47,48].

A subset of AVMs occurs in the context of hereditary conditions, most notably HHT, an autosomal dominant disorder caused by mutations in *ENG*, *ACVRL1*, or *SMAD4* [11,19,25,49,50,51]. These patients frequently harbor multiple AVMs in the lungs, liver, and brain, requiring multidisciplinary surveillance and long-term management. Another well-described syndrome is capillary malformation–arteriovenous malformation (CM-AVM), caused by mutations in *RASA1* (RAS p21 protein activator 1) and *EPHB4* (Ephrin type-B receptor 4), characterized by multifocal fast-flow lesions and cutaneous vascular malformations [27,49,51]. Clinical presentation varies by anatomic location, nidus size, and flow characteristics. For cAVMs, intracranial hemorrhage is the most common presentation, occurring in ~50% of cases [26,52,53]. Hemorrhages are typically intraparenchymal but may extend into subarachnoid or intraventricular compartments, with seizures occuring in 20–40% of patients, particularly with cortical involvement [10,26]. Other presentations include chronic headaches, focal neurological deficits, or progressive decline related to venous hypertension and vascular steal in eloquent brain regions [10,36].

The annual risk of bleeding for unruptured cAVMs is ~2–4% but rises to 6–15% following a first hemorrhage [1,47,54]. These data emphasize the need for individualized risk assessment, particularly in asymptomatic patients under surveillance [46,47,50]. Spinal AVMs, while less common, produce distinct clinical features such as progressive myelopathy, radicular pain, or acute neurological decline due to ischemia or venous hypertension [35].

Extracranial AVMs display a wide spectrum of presentations [4,11,25,50,55]. Pulmonary AVMs, often seen in HHT, may cause hypoxemia, paradoxical embolism, or stroke [11,50,56,57]. Gastrointestinal AVMs can present occult bleeding or chronic anemia, while hepatic AVMs may lead to high-output cardiac failure or portal hypertension [57]. Musculoskeletal and soft tissue AVMs often produce localized pain, swelling, limb overgrowth, or impaired function [12,13,35]. In some cases, extensive arteriovenous shunting can cause systemic complications, such as increased cardiac preload or ischemia of adjacent tissues [1,57,58,59].

Given this heterogeneity, timely diagnosis and appropriate management require multidisciplinary expertise. Syndromic AVMs necessitate multisystem surveillance, while isolated AVMs benefit from targeted imaging, regular monitoring, and, when appropriate, intervention. Future integration of genetic insights with detailed clinical phenotyping may further enhance diagnostic precision and guide individualized treatment strategies.

## 3. Pathogenesis and Molecular Mechanism

AVMs result from complex disturbances in vascular development and remodeling, influenced by both genetic and acquired factors [5]. Although typically congenital, AVMs are increasingly recognized as dynamic lesions modulated by molecular and hemodynamic cues throughout life [10,43,52,60,61,62]. At the cellular level, aberrant arteriovenous connections arise from disruptions in endothelial specification, vessel patterning, and mural cell recruitment [56,58,62,63,64]. A central mechanism is the failure of vascular stabilization and remodeling, rather than complete developmental arrest, suggesting that AVMs may progress postnatally under permissive conditions such as inflammation, injury, or hormonal stimulation [28,58,62,63].

A major pathway implicated in AVM biology is the TGF-β superfamily, particularly *ENG* and *ACVRL1* [63]. Both are essential for maintaining endothelial quiescence and vascular integrity [18,38,63,65,66,67]. Mutations in these genes, as observed in HHT, impair angiogenic regulation and drive abnormal proliferation and vessel dilation [11,25,50,56]. Genetic defects alone, however, are insufficient to produce AVMs. In animal models, localized angiogenic stimuli such as VEGF overexpression are required to trigger malformation, supporting a “two-hit” model of disease [19,53,56,64].

Importantly, the molecular mechanisms driving AVM formation differ between endothelial cells (ECs) and vascular smooth muscle cells (VSMCs) [68,69,70]. In ECs, mutations in TGF-β pathway genes (*ENG*, *ACVRL1*), as well as activation of RAS/MAPK, PI3K/AKT, and Notch signaling, primarily disrupt endothelial quiescence, arterial–venous specification, and angiogenic regulation [70,71]. These alterations lead to abnormal endothelial proliferation, arteriovenous shunting, and formation of AVM nidus [68,70,71]. Similarly, VSMCs rely on TGF-β signaling to maintain differentiation, vessel wall integrity, and extracellular matrix deposition [69,70]. Defective VSMC signaling results in reduced mural cell coverage and weakened vessel support, amplifying vascular fragility and predisposing AVMs to dilation and hemorrhage [69,71]. Together, these cell-specific mechanisms illustrate how aberrant EC behavior establishes the malformed vascular network while impaired VSMC function fails to stabilize it, synergistically driving AVM pathogenesis [71]. The relationship between endothelial cells and vascular smooth muscle cells remains markedly understudied in the context of AVM pathogenesis, which warrants the need for future in-depth investigations to clarify their distinct and coordinated contributions.

In sporadic AVMs, somatic mutations have been identified in signaling genes, including *KRAS*, *MAP2K1*, and *PIK3CA* [20,25,72]. These mutations activate RAS/MAPK and PI3K/AKT signaling, leading to unchecked endothelial proliferation and aberrant angiogenesis [25]. Importantly, *KRAS* mutations have been found in endothelial cells of human brain AVMs, providing the first direct evidence that sporadic lesions may result from postzygotic mosaic mutations [20,25,72]. This paradigm mirrors other vascular malformations and overgrowth syndromes [5,54,60]. Such discoveries highlight new therapeutic opportunities, with *MEK* inhibitors showing promise in preclinical RAS-mutated AVM models [20,64].

Notch signaling also plays a crucial role in arterial–venous specification and branching morphogenesis [64,68,73,74,75,76]. Dysregulation of Notch, particularly Notch4, induces arteriovenous shunting and vessel enlargement in murine models [18,20,28,77,78,79,80]. Endothelial-specific overexpression of activated Notch4 generates malformations resembling human AVMs, independent of VEGF signaling [18,28,42,64,77,81,82]. These findings suggest Notch serves as a convergence point for developmental and pathological vascular remodeling [83]. Furthermore, reduced mural cell coverage and extracellular matrix components in AVM nidus tissue contribute to vascular fragility and hemorrhagic risk [20,84].

Beyond genetics, biomechanical forces and inflammation influence AVM initiation and progression [21,85]. High shear stress and disturbed flow activate endothelial transcriptional programs that promote angiogenesis and remodeling, particularly when TGF-β or Notch signaling is impaired [53,68,86,87]. Inflammatory mediators such as TNF-α and IL-6 are elevated in AVM tissue and further amplify endothelial proliferation, leukocyte recruitment, and extracellular matrix degradation, exacerbating lesion instability [53,85,86,88].

Brain AVMs (bAVMs) are abnormal high-flow tangles of arteries and veins that bypass the capillary network due to disrupted vascular development and defective arterial–venous specification, resulting in fragile vessels with a high risk of intracranial hemorrhage [36,89,90]. Brain AVMs arise primarily from developmental failures in arterial–venous specification and neurovascular unit regulation, leading to chaotic, fragile vascular networks directly influenced by abnormal endothelial identity, disrupted signaling (RAS/MAPK, NOTCH, TGF-β), and impaired interactions with astrocytes and pericytes [36,91,92]. In contrast, peripheral AVMs, those found in other vascular beds, more commonly result from reactive pathological angiogenesis driven by injury, ischemia, inflammation, and abnormal hemodynamic forces, with vessel remodeling shaped by VEGF-dominant signaling, stromal fibrosis, and adaptive structural changes rather than intrinsic defects in vascular identity [3,21,45]. The differentiation between bAVM and peripheral AVM can be seen in Table 1.

Moreover, studies have provided increasing evidence that non-coding RNAs, especially microRNAs (miRNAs), exert significant regulatory influence over the signaling pathways implicated in bAVM pathogenesis [89,90]. Several miRNAs are markedly dysregulated in both AVM tissue and vascular smooth muscle cells, including downregulation of miR-18a, miR-137, and miR-195*, and upregulation of miR-7-5p, miR-199a-5p, miR-200b-3p, and the let-7 family. These alterations influence established vascular signaling pathways, particularly TGF-β/SMAD, VEGF, and inflammatory cascades, by targeting genes that govern endothelial quiescence, extracellular matrix remodeling, and angiogenic activity [89]. For example, decreased miR-18a may contribute to excessive VEGF expression and destabilization of vascular architecture, while reduced miR-137 and miR-195* promote smooth muscle phenotypic switching and inflammation, collectively contributing to AVM-like vessel behavior [89].

Complementing these findings, the usage of applied machine-learning analysis to identify circulating and tissue miRNAs that map onto pathogenic network is highly relevant to AVM formation [90]. Their computational modeling linked the upregulated miRNAs, such as miR-7-5p, miR-199a-5p, miR-200b-3p, and let-7b-5p, to pathways including RAS/MAPK, PI3K/AKT, and VEGF-driven endothelial proliferation [90]. Meanwhile, downregulated miR-18a and related miRNAs were associated with dysregulation of TGF-β-dependent vascular stability signals. Together, these data suggest that miRNAs act as upstream modulators of multiple converging pathways that drive pathological angiogenesis, endothelial hyperproliferation, and vessel fragility in bAVMs [90]. Integrating non-coding RNA biology into the molecular framework of AVMs therefore offers a more complete understanding of disease mechanisms and highlights promising avenues for biomarker development and therapeutic targeting [90]. Taken together, these insights reinforce AVMs as multifactorial lesions shaped by intertwined genetic, molecular, and biomechanical forces, underscoring the need for integrated, pathway-directed therapeutic strategies [89,90].

**Figure 2:**
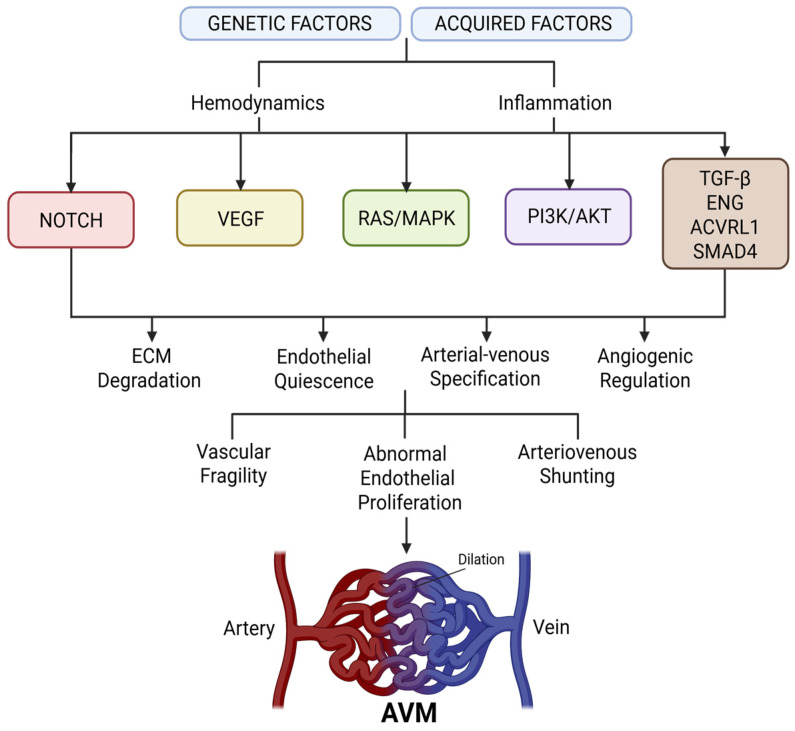
Molecular mechanisms contributing to arteriovenous malformation (AVM) formation. The schematic illustrates how both genetic and acquired factors converge on key endothelial signaling pathways that regulate vascular development and remodeling. Mutations or dysregulation of pathways disrupt endothelial cell specification, angiogenic balance, and vessel stabilization. These perturbations collectively impair arterial–venous identity, promote aberrant endothelial proliferation and migration, and weaken mural cell recruitment. The resulting failure of normal vascular maturation leads to the development of a direct arteriovenous connection, depicted as a tangled nidus lacking an intervening capillary bed, establishing the hallmark features of AVMs.

Taken together, AVM pathogenesis reflects a multifactorial process involving germline and somatic mutations, dysregulated angiogenic signaling, aberrant mechanotransduction, and inflammatory responses (Figure 2) [2,21,36,81,82]. Advances in molecular profiling have refined understanding of these mechanisms and uncovered new targets for pharmacologic intervention [2,21,50]. As precision medicine evolves, integration of genetic diagnostics and pathway-specific therapies offers the potential for individualized management, especially in surgically inaccessible or refractory lesions.

## 4. Diagnosis and Treatment

The clinical presentation of AVMs at the time of diagnosis is strongly determined by anatomical location, size, and vascular dynamics, resulting in significant heterogeneity across organ systems. Within the central nervous system (CNS), patients may present with seizures, focal neurological deficits, chronic headaches, or intracranial hemorrhage, depending on the lesion’s proximity to eloquent brain regions [2,3,5,36,91,92]. AVMs located in the gastrointestinal tract frequently manifest chronic gastrointestinal bleeding, anemia, abdominal pain, or, in severe cases, high-output cardiac failure due to large-volume arteriovenous shunting [21,40,54,93]. Musculoskeletal AVMs may cause localized pain, tissue overgrowth, venous congestion, deformities, or limb hypertrophy, often progressing to functional impairment if untreated [2,3,5,14,53]. Pulmonary AVMs, commonly associated with HHT, can lead to hypoxemia, paradoxical embolization, and cryptogenic stroke [19,25,56]. Conversely, minorities of the AVMs are diagnosed incidentally and remain asymptomatic. Recognizing these diverse presentations is crucial for timely and accurate diagnosis.

In addition to overt clinical symptoms, subclinical or small AVMs may remain asymptomatic for years, only being discovered incidentally during imaging for unrelated conditions [13]. These incidental findings present diagnostic dilemmas, as their natural history and rupture risk can vary substantially [94]. Syndromic associations, such as HHT or capillary malformation–AVM syndrome (linked to *RASA1* and *EPHB4* mutations), further complicate diagnosis, as multiple lesions in different organs may coexist [27,29,54,95]. A high index of suspicion is therefore required in patients with recurrent epistaxis, mucocutaneous telangiectasias, or a family history of AVM-related disorders.

Accurate diagnosis relies on a multimodal imaging approach, with each modality offering unique insights into lesion architecture and hemodynamics. Digital subtraction angiography (DSA) remains the gold standard for AVM characterization, providing dynamic, high-resolution visualization of arterial feeders, the nidus, and venous drainage patterns [36,54,60,96,97]. However, given its invasive nature, DSA is typically reserved for definitive evaluation and treatment planning.

Magnetic resonance imaging (MRI) has become the most widely used noninvasive modality, particularly for CNS AVMs [31]. T1- and T2-weighted sequences demonstrate flow voids, while susceptibility-weighted imaging (SWI) detects small hemorrhages or microbleeds [34]. Perfusion-weighted MRI and MR angiography provide hemodynamic information, such as shunting and regional perfusion deficits [34]. High-field 7T MRI is under investigation for its ability to detect micro-AVMs and refine assessment of cortical involvement [34].

Computed tomography (CT) and CT angiography (CTA) are invaluable for rapid assessment in the acute setting, particularly when hemorrhage is suspected [2,60,96]. CTA allows reconstruction of vascular networks and can delineate nidus size and draining veins in patients who are unstable for MRI or angiography [60].

Ultrasonography, particularly color Doppler and duplex ultrasound, offers a real-time, inexpensive, and radiation-free modality for superficial AVMs [31]. It is often used in pediatric or peripheral lesions. Three-dimensional rotational angiography (3D-RA) represents a major advancement, providing unparalleled spatial resolution for surgical or endovascular planning [31,43,98].

Emerging modalities include positron emission tomography (PET), which may detect metabolic activity within the nidus, and molecular imaging strategies targeting angiogenesis-related markers [2,60,96]. Artificial intelligence (AI) and machine learning are also being investigated for automated AVM segmentation, rupture-risk prediction, and treatment planning, offering potential to reduce interobserver variability [53,60,96,97].

Small, asymptomatic, or deeply located AVMs are often missed with conventional imaging. These lesions may present only with subtle symptoms or be masked by coexisting vascular abnormalities [60,92,96]. Contrast-enhanced imaging or serial studies are sometimes required to capture dynamic changes in vascular flow [54,60,96,97]. In syndromic patients with multisystem disease, whole-body imaging or targeted screening may be required to identify extracranial lesions [54,60,91,96,97].

Advancements in genomics and biomarker discovery are expected to augment imaging-based diagnostics. Identifying circulating biomarkers (e.g., endothelial dysfunction proteins, angiogenic factors, or cell-free DNA carrying *KRAS*/*PIK3CA* mutations) could help detect subclinical AVMs and stratify rupture risk [27,72,87,99]. Integration of genomics with imaging will enable precision diagnostics in linking AVM morphology to underlying molecular drivers.

**Figure 3:**
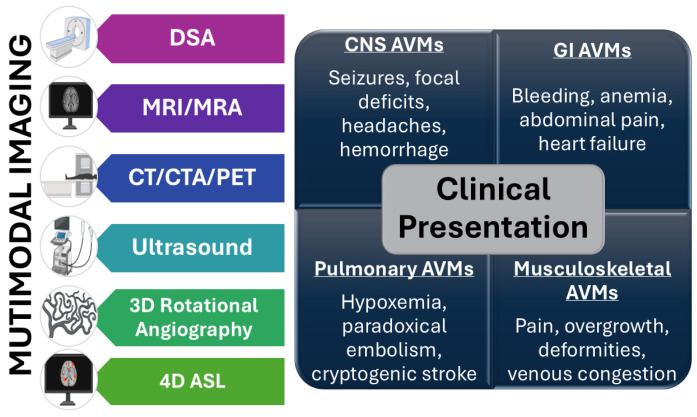
AVM diagnosis requires a tailored, patient-specific approach that integrates clinical evaluation with multimodal imaging, including digital subtraction angiography (DSA), magnetic resonance imaging and magnetic resonance angiography (MRI/MRA) with perfusion and susceptibility sequences, computed tomography angiography (CTA), Doppler ultrasound, 3D rotational angiography, arterial spin labeling (ASL), and 4D flow techniques refining hemodynamic assessment.

In summary, AVM diagnosis requires a tailored, patient-specific approach that integrates multimodal imaging with careful clinical evaluation (Figure 3). While DSA remains the gold standard, complementary use of MRI, CTA, ultrasound, and advanced imaging enhances diagnostic accuracy. Despite these advances, detecting small or asymptomatic AVMs continues to pose challenges. Future strategies combining imaging, genomics, and computational modeling promise earlier detection, individualized risk assessment, and improved treatment planning.

## 5. Current Treatment Options

### 5.1. Embolization, Surgery, and Radiation Therapy

**Figure 4:**
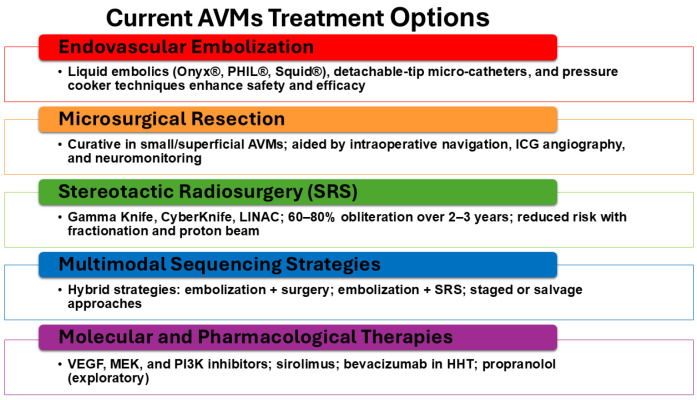
Current treatment strategies for AVMs. Optimal AVM management increasingly relies on multimodal sequencing strategies that integrate endovascular, surgical, and radiosurgical approaches. Endovascular embolization may be used preoperatively to reduce flow and nidus size, as an adjunct to stereotactic radiosurgery (SRS), or as a stand-alone therapy in select lesions. Microsurgical resection remains the most definitive curative option for accessible AVMs, particularly in low-grade lesions, but carries risks of hemorrhage and neurological morbidity. Stereotactic radiosurgery provides a non-invasive alternative for small or surgically inaccessible AVMs, though obliteration may take years, and hemorrhage risk persists during the latency period. Hybrid and staged strategies, such as embolization followed by surgery or SRS, can maximize efficacy and minimize complications, especially for large or complex AVMs. Salvage approaches are employed when recurrence or incomplete obliteration occurs. Together, these treatment modalities highlight the importance of individualized, multidisciplinary planning to balance cure, risk reduction, and functional outcomes.

The therapeutic management of AVMs is complex, requiring careful assessment of patient-specific and lesion-specific factors to determine the safest and most effective intervention (Figure 4) [21]. Historically, treatment relied on surgical excision or supportive care, but modern advances in endovascular techniques, microsurgical technologies, and stereotactic radiosurgery (SRS) have transformed outcomes [12,47]. No single modality universally applies to all AVMs; instead, management strategies are tailored according to nidus size, venous drainage, eloquence of involved tissue, and hemorrhagic presentation [3,5,14,36,92].

### 5.2. Endovascular Embolization

Embolization has evolved into a pivotal therapeutic and adjunctive modality. Early embolization employed particulate agents or alcohol, which were associated with high recurrence and complication rates. The introduction of n-butyl cyanoacrylate (NBCA) marked the first major improvement, enabling deeper penetration of the nidus [5]. In the past two decades, newer liquid embolics, such as Onyx^®^ (ethylene-vinyl alcohol copolymer), PHIL^®^ (precipitating hydrophobic injectable liquid), and Squid^®^, have significantly improved treatment durability by permitting controlled injections and deeper nidus occlusion [3,100].

Technical refinements, including detachable-tip microcatheters, dual-lumen balloon catheters, and the “pressure cooker technique,” have improved precision and minimized reflux-related complications [60,100]. Cone-beam CT and 3D rotational angiography now permit real-time navigation, enabling targeted embolization of deep feeders that were previously inaccessible [31].

While curative embolization is feasible for some small AVMs, it is most often used adjunctively to reduce nidus size, decrease flow, and prepare patients for subsequent microsurgery or radiosurgery [100]. Reported obliteration rates with embolization remain variable, ranging from 10% to 50% depending on AVM architecture and embolic agent used [100]. Complications include inadvertent venous occlusion, ischemia, and hemorrhage, underscoring the need for experienced multidisciplinary teams.

### 5.3. Microsurgical Resection

Surgical excision remains the only definitive curative therapy for appropriately selected patients [17,100,101]. The Spetzler-Martin grading system has long guided selection, balancing surgical risk against potential benefit. Advances in microsurgical techniques, including intraoperative neuronavigation, indocyanine green (ICG) angiography, and continuous neuromonitoring, have enhanced safety and completeness of resection [17,101].

For small, superficial AVMs located in non-eloquent regions, microsurgical resection achieves obliteration rates exceeding 95% with low morbidity [101]. However, for deep-seated or high-grade AVMs, surgical risks increase significantly, with permanent neurological deficit rates reported at 10–20% in some series [101]. To mitigate this, hybrid approaches incorporating preoperative embolization to reduce intraoperative bleeding are now standard practice in many centers [100,101].

### 5.4. Stereotactic Radiosurgery (SRS)

SRS has become a cornerstone for the management of small- to medium-sized AVMs (<3 cm) or those located in eloquent or surgically inaccessible areas. Platforms such as Gamma Knife, CyberKnife, and LINAC-based systems deliver precise, high-dose radiation to the nidus, inducing gradual endothelial proliferation, luminal occlusion, and obliteration over 2–3 years [17]. Obliteration rates range from 60 to 80% at 3 years, with hemorrhage risk decreasing during the latency period [5,20,53].

Complications include radiation necrosis, edema, and delayed cyst formation, but improved dose planning, fractionated SRS, and proton beam therapy are reducing such risks [3]. Combining embolization and radiosurgery, particularly staged embolization followed by SRS, has shown promise in improving obliteration rates for larger or complex AVMs [2,14,100].

### 5.5. Multimodality and Sequencing Strategies

No single modality is universally curative; therefore, integrated multimodal strategies are increasingly emphasized [22,34,60]. Embolization followed by microsurgery reduces intraoperative bleeding and operative time [40]. Radiosurgery following partial embolization is used for residual nidus reduction, while surgery following failed SRS provides salvage therapy. Emerging “hybrid operating rooms,” which combine angiographic and neurosurgical capabilities, enable intraoperative angiography and immediate resection following embolization, reducing recurrence risk [22].

### 5.6. Emerging Minimally Invasive and Endovascular Interventions

New endovascular technologies are shifting the treatment landscape. Flow-diverting stents and covered stents are under investigation for redirecting arterial inflow, though risks of hemorrhage remain a concern [21]. Bioactive coils and liquid embolics with radiopaque markers are improving visualization and precision [60]. Advanced intraoperative imaging, including flat-panel CT and fusion angiography, enhances nidus targeting [60].

### 5.7. Novel Molecular and Pharmacologic Therapies

Conventional mechanical approaches are now being supplemented by pharmacologic interventions targeting dysregulated signaling pathways. VEGF inhibitors, MEK inhibitors (for *KRAS*/*MAP2K1* mutant AVMs), PI3K inhibitors (for *PIK3CA* mutant lesions), and sirolimus (mTOR inhibition) are under active investigation [29,59,99,102]. Case series in HHT have reported reduced epistaxis and AVM stabilization with bevacizumab, suggesting systemic therapy could serve as an adjunct to conventional modalities [3]. Propranolol, widely used for infantile hemangiomas, has also been explored for AVMs with anecdotal benefit [2,53].

### 5.8. Animal Models and Translational Insights

Animal models remain indispensable for understanding AVM biology and testing novel interventions (Table 1). Genes potentially associated with AVM formation are listed in Table 2. Genetically engineered mice with *ENG*, *ALK1*, or *RASA1* mutations recapitulate HHT-associated AVMs and have revealed key roles for TGF-β, Notch, and VEGF pathways (Table 3) [1,2,3,4,19,24,27,42,49,52,54,56,64,87,88,89,94,103,104,105,106,107]. Zebrafish models allow high-throughput screening of angiogenesis inhibitors, while optogenetic vascular models permit dynamic modulation of flow and vessel remodeling [41]. Preclinical successes are guiding translation trials of *MEK* inhibitors and anti-angiogenic therapies [108].

The future of AVM management lies in integrating genomics, imaging, and novel therapies into precision medicine frameworks. AI-driven rupture-risk prediction, imaging-based treatment planning, and molecular-targeted therapy are expected to complement surgical, endovascular, and radiosurgical modalities. Bioengineering approaches, including vascular tissue scaffolds and regenerative strategies, may one day enable reconstruction of normal vasculature following AVM obliteration.

### 5.9. Challenges in Developing Effective Treatments for AVMs and Future Directions

Despite significant advances in AVM therapies, several critical challenges persist. The heterogeneity of AVMs with differences in size, location, venous drainage, and clinical symptoms necessitates individualized, case-specific strategies [3,53,54,100]. Even after technically successful interventions, recurrence or incomplete nidus obliteration is not uncommon, highlighting the need for lifelong surveillance with advanced imaging modalities such as high-field MRI and catheter angiography [60,97]. Another unresolved issue is the optimal sequencing of treatment modalities, particularly when combining embolization, microsurgery, and radiosurgery [2,3,21,31,93,101]. Refinements in targeted molecular therapies, the development of novel embolic agents, and the integration of advanced imaging-guided planning represent promising avenues for reducing recurrence rates and improving long-term safety [31,60,100]. At the same time, ongoing research into the genetic and molecular underpinnings of AVMs continues to uncover new therapeutic targets [3].

The comprehensive management of AVMs demands a multidisciplinary team approach, involving neurosurgeons, interventional neuroradiologists, neurologists, and radiation oncologists. Vascular surgeons often play a role in extracranial AVMs, while anesthesiologists with neurovascular expertise contribute to intraoperative safety. Imaging specialists are crucial for assessing nidus morphology and hemodynamics, which in turn informs treatment planning [22,110]. This collaborative framework ensures holistic, patient-centered care that considers not only procedural safety but also comorbidities and quality of life [23,36,60].

Long-term follow-up remains essential for evaluating treatment efficacy and patient outcomes. While successful intervention may result in AVM obliteration, residual risks such as delayed hemorrhage, treatment-related neurological deficits, and AVM recurrence persist [2,3,31,53,54,91]. Pediatric and young adult patients are particularly vulnerable, as recurrence has been documented even after apparent cure [20,61]. Structured longitudinal assessments provide insights into the durability of treatment outcomes and inform best practices for surveillance. Including patients that are asymptomatic that were diagnosed incidentally. Consequently, the decision to treat these patients is also complex because this depends on the anatomical and physiological features of the AVM and would involve a multidisciplinary approach.

Patient education and engagement are equally critical. Patients and families should receive clear information regarding natural history, treatment risks, and the necessity of routine follow-up. Scheduled imaging (e.g., MRI/MRA, angiography) is central to detecting recurrence or residual lesions [3,36,54]. Empowering patients through education fosters proactive care-seeking behaviors, facilitates adherence to monitoring protocols, and enhances long-term quality of life.

While substantial progress has been made in elucidating AVM biology, unanswered questions remain. The genetic and environmental factors that predispose individuals to AVM development are incompletely understood [46]. Although somatic mutations in genes such as *KRAS*, *MAP2K1*, and *PIK3CA* have been implicated in sporadic AVMs, the precise triggers initiating lesion progression remain elusive [1,15,20,63,72,109]. Future research must unravel the interplay of angiogenic signaling, hemodynamic stress, and inflammation in nidus formation and rupture.

Looking forward, the integration of advanced imaging and genomics is expected to refine diagnosis and treatment selection. High-resolution modalities such as 7T MRI and 4D flow angiography can improve characterization of AVM morphology and hemodynamics [15,20,72]. Concurrently, genomic profiling holds promise for identifying biomarkers predictive of rupture risk or treatment response [1,15]. Together, these advances enable more precise risk stratification and lay the foundation for personalized treatment paradigms tailored to the individual patient.

Monitoring represents a critical component in the management of AVMs, given their dynamic nature and variable risk of hemorrhage or neurological compromise [3]. Regular imaging follow-up with MRI, CT angiography, or digital subtraction angiography allows clinicians to track lesion growth, assess hemodynamic changes, and identify early signs of instability or recurrence, particularly after intervention, especially in asymptomatic patients [36]. The choice of treatment must be individualized, considering lesion size, location, vascular architecture, patient age, comorbidities, and rupture risk [3,36,92]. Therapeutic options range from microsurgical resection, endovascular embolization, and stereotactic radiosurgery to combination approaches, each with distinct efficacy and risk profiles [23,36]. By integrating careful monitoring with individualized treatment strategies, clinicians can optimize outcomes while minimizing procedural risks, especially in surgically challenging or recurrent lesions.

The emergence of precision medicine approaches is further reshaping AVM care. Tailoring therapy to individual lesion characteristics includes size, location, and genetic or molecular profile, which has the potential to improve outcomes while minimizing morbidity [1,15]. Pharmacologic interventions targeting VEGF, Notch, or PI3K/AKT signaling are under active investigation [109]. Integrating such strategies with surgical and endovascular care may optimize long-term disease control. Collectively, combining advanced imaging, genomic insights, and precision therapeutics offers a holistic framework for personalized AVM management. Ultimately, such an integrated approach bridges mechanistic understanding with clinical practice, paving the way for safer, more effective, and individualized AVM therapies [3,93].

Finally, ethical considerations are central to advancing AVM research and care. Ensuring informed consent, transparency about risks, and equitable access to novel therapies remain essential. As new technologies emerge, including molecular therapies and AI-based rupture-risk prediction, ongoing ethical oversight will be critical to balance innovation with patient safety.

In summary, AVMs represent a multifaceted clinical challenge. Continued exploration of their genetic basis, coupled with innovations in imaging, targeted therapies, and multidisciplinary management, offers hope for improved outcomes. By advancing both biological understanding and therapeutic technologies, the field is moving toward more effective, durable, and personalized solutions for patients with these complex vascular anomalies.

## 6. Conclusions

AVMs arise from a multifaceted interplay of genetic, molecular, and hemodynamic factors that govern their initiation, progression, and clinical complications. Advances in imaging technologies and molecular diagnostics have greatly enhanced the ability to detect and characterize AVMs, thereby improving risk stratification and guiding individualized treatment planning. Despite progress with conventional therapies such as endovascular embolization, microsurgical resection, and stereotactic radiosurgery, significant challenges remain, including recurrence, incomplete obliteration, and treatment-related morbidity. The involvement of dysregulated signaling pathways, notably Notch, VEGF, and TGF-β, underscores the need for targeted molecular therapies that can directly address abnormal angiogenesis and vascular remodeling. In parallel, computational modeling and experimental research continue to shed light on AVM hemodynamics, opening new avenues for therapeutic innovation.

Future research must prioritize the refinement of robust preclinical models that faithfully replicate the heterogeneity of human AVMs. Genetically engineered mouse models, zebrafish platforms, and other in vivo systems have already yielded critical insights, yet further optimization is required to enhance translational relevance. These models will remain indispensable for dissecting pathway-specific mechanisms and for testing novel pharmacologic approaches, such as inhibitors of aberrant Notch or VEGF signaling, which may ultimately provide less invasive and more durable treatment options.

Equally important is the integration of genomics, biomarker discovery, and personalized medicine into both research and clinical practice. Advances in bioengineering and regenerative medicine hold promises for vascular remodeling strategies that could complement or replace conventional interventions. A truly multidisciplinary framework uniting neurosurgeons, interventional radiologists, geneticists, vascular biologists, biomedical engineers, and computational scientists will be critical to translate emerging scientific insights into patient care. By leveraging these collaborative advances, the field can move toward precision therapies that improve outcomes, reduce recurrence, and ultimately lessen the burden of AVM-related morbidity and mortality.

## Figures and Tables

**Table 1:** Comparative Features of Brain AVMs versus Peripheral AVMs.

Feature	Brain AVMs	Peripheral AVMs
Primary Origin	Developmental/congenital defect in vascular patterning	Congenital or frequently acquired (trauma, inflammation, ischemia)
Key Mechanism	Loss of arterial–venous identity and neurovascular unit dysfunction	Pathologic angiogenesis driven by injury and hemodynamic stress
Dominant Pathways	Developmental dysregulation: RAS/MAPK, NOTCH, TGF-β, VEGF	Reactive angiogenesis: VEGF and TGF-β
Vessel Structure	Fragile, thin walled, poorly supported vessels	More fibrotic, remodeled, adaptive vessels
Flow Pattern	High-flow chaotic nidus	Often localized fistula-like shunts
Inflammation	Neuroinflammatory (microglia-dominant)	Chronic peripheral inflammation (macrophage-dominant)
Hemorrhage Risk	High–frequent intracranial bleeding	Lower (except pulmonary AVMs)
Unique Feature	Involves blood–brain barrier and neurovascular coupling	Involves connective tissue and stromal remodeling

**Table 2:** Genetic and Mechanistic Overview of Arteriovenous Malformation in Mouse Models.

Pathway	Representative Mouse Models	Mechanistic Basis	Brain AVM Incidence	Non-Brain AVM Incidence	Major Phenotypes	Key Insights/Relevance	Ref.
TGF-β/ALK1/ENG Pathway	Eng^+/−^, Alk1^+/−^, Eng^fx/fx^ (endothelial or smooth muscle Cre), Alk1^fx/fx^ (L1-Cre, SM22α-Cre), Eng^+/−^ + VEGF, Alk1^fx/fx^ + AAV-VEGF	Loss of endothelial TGF-β signaling (via ENG or ALK1) impairs flow-dependent vascular remodeling, leading to persistence of arteriovenous shunts.	Moderate to high (25–100% depending on deletion timing and VEGF stimulation)	Occasional (skin, lung, liver) especially with smooth muscle Cre lines	Dilated and tortuous vessels, arteriovenous shunts, hemorrhage, neurological dysfunction, lethality (in embryonic or severe adult deletion)	Core pathway in hereditary hemorrhagic telangiectasia (HHT); demonstrates requirement of ENG/ALK1 for vessel quiescence; adult angiogenesis triggers AVMs only with combined deletion + VEGF	[16,19,20,56,64,94,104,105,106]
Notch Signaling Pathway	Tie2-tTA; TRE-Notch4 or Notch1 (gain-of-function), Cdh5(PAC)-CreERT2; Rbpj^fx/fx^ (loss-of-function)	Constitutive activation of Notch signaling forces arterial identity and disrupts arterial–venous segregation; Rbpj deletion blocks canonical Notch signaling.	Very high (≈100% when activated early in development)	Yes—lesions also in liver, skin, uterus with sustained activation	Enlarged and tortuous vessels, high-flow AV shunts, hemorrhage, neurological signs, lethality in early activation	Demonstrates that Notch activation alone is sufficient to induce AVMs; phenotype is reversible upon Notch4 suppression; cross-talk with ALK1/TGF-β pathways critical for lesion persistence	[64,74,105,107]
BMP/Mgp-Related Pathway	Mgp^−/−^ (Matrix Gla Protein knockout)	Loss of BMP inhibition leads to upregulation of BMP/ALK1/Notch signaling, causing abnormal endothelial activation and arteriovenous connections.	High (≈100%)	Yes (skin, heart, lung, kidney)	AV shunts, vessel dilation, hemorrhage, early lethality	Links vascular calcification genes with AVM pathogenesis; supports shared downstream effectors with ALK1/ENG and Notch pathways	[64,104,109]
Combined Pathways/Cross-Talk Models	Mgp^−/−^ + Jag1^+/−^, Eng^+/−^ + VEGF, Alk1^+/−^ + VEGF, Eng^fx/fx^ + AAV-VEGF	Interaction between VEGF-driven angiogenesis and impaired ENG/ALK1/Notch signaling promotes AVM initiation and maintenance.	High (up to 90%)	Yes (skin, ear, visceral organs)	Robust angiogenesis, nidus formation, hemorrhage, regression with VEGF withdrawal or pathway restoration	Demonstrates that angiogenic context is essential for AVM formation; provides model for adult-onset or acquired AVMs	[64,96,106,108]

**Table 3:** Genes Implicated in AVM Formations.

Category	Representative Genes	Ref.
Angiogenic/Vascular Signaling	ENG, ALK1 (ACVRL1), GDF2, RASA1, PDGFB, EFNA4, SLIT2, NOTCH	[1,2,3,4,27,42,49,52,59,63,96]
ECM/Structural	TNXB, CSPG4, CD109, FLRT3	[87,88,97]
Growth Factor/Signaling Modulators	TGF-β/SMAD, COUP-TFII, SOX18, PROX1, NFATc1, FOXC2, VEGF	[3,45,88,97]
Miscellaneous/Emerging	NBPF10, NAXE, TTC21B, BMP3, IGFBP7, AOC3, NEDD4L, STK4, MAP2K1	[1,45,68,88]

## Data Availability

No new data was generated from this article.

## Abbreviations

The following abbreviations are used in this manuscript:

3D-RA	Three-Dimensional Rational Angiography
ACVRL1	Activin Receptor-like Kinase
AI	Artificial Intelligence
AVM(s)	Arteriovenous Malformation(s)
bAMV(s)	Brain Arteriovenous Malformation(s)
cAVM(s)	Cerebral Arteriovenous Malformation(s)
CM-AVM	Capillary malformation–Arteriovenous Malformation
CNS	Central Nervous System
CT	Computed Tomography
CTA	Computed Tomography Angiography
DSA	Digital Subtraction Angiography
EC(s)	Endothelial Cell(s)
EPH4	Ephrin type-B receptor 4
ENG	Endoglin
HHT	Hereditary Hemorrhagic Telangiectasia
ICG	Indocyanine Green
KRAS	Kirsten Rat Sarcoma Virus
MAP2K1	Mitogen-activated Protein Kinase Kinase 1
MRI	Magnetic Resonance Imaging
NBCA	N-Butyl Cyanoacrylate
PET	Positron Emission Tomography
PIK3CA	Phosphatidylinositol-4,5-Bisphosphate 3-Kinase Catalytic Subunit Alpha
RASA1	RAS p21 protein activator 1
SMAD4	Mothers Against Decapentaplegic Homolog 4
SWI	Susceptibility-Weighted Imaging
TGF-β	Transforming Growth Factor–β
VEGF	Vascular Endothelial Growth Factor
VSMC(s)	Vascular Smooth Muscle Cell(s)
